# Natural and engineered charge-transfer and diradical state mixing and retinal chromophore dynamics

**DOI:** 10.1039/d6cp02043g

**Published:** 2026-09-24

**Authors:** Massimo Olivucci, Michael A. Robb

**Affiliations:** a Chemistry Department, University of Siena Via Aldo Moro n. 2 53100 Siena Italy massimo.olivucci@unisi.it; b Chemistry Department, Bowling Green State University Overman Hall 43403 OH USA; c Department of Chemistry, Molecular Sciences Research Hub, Imperial College London White City Campus, 80 Wood Lane London W12 0BZ UK mike.robb@imperial.ac.uk

## Abstract

Recent experimental and computational results show that a second singlet electronic state may be transiently populated during the photoisomerization of retinal chromophores. Such a state is dominated by a doubly excited electronic configuration, and it is qualitatively different from the spectroscopically allowed state featuring a dominant singly excited configuration and charge-transfer character. In this contribution, we review and discuss the consequences of the mixing of these two states on the photoinduced dynamics of different models of the retinal chromophore. In doing so, we consider the results of simulations carried out using quantum-classical trajectories driven by Born–Oppenheimer forces, as well as using methods where both electron and nuclear motions are simultaneously propagated with the quantum Ehrenfest approach. We conclude that electronic state mixing is a viable strategy for modulating the retinal chromophore dynamics and reactivity in both natural and artificial systems.

## Abstract 2

Recent experimental and computational results show that a second singlet (S_2_) electronic state may be transiently populated during the early phase of the photoisomerization dynamics of retinal chromophores.

## Graphical abstract

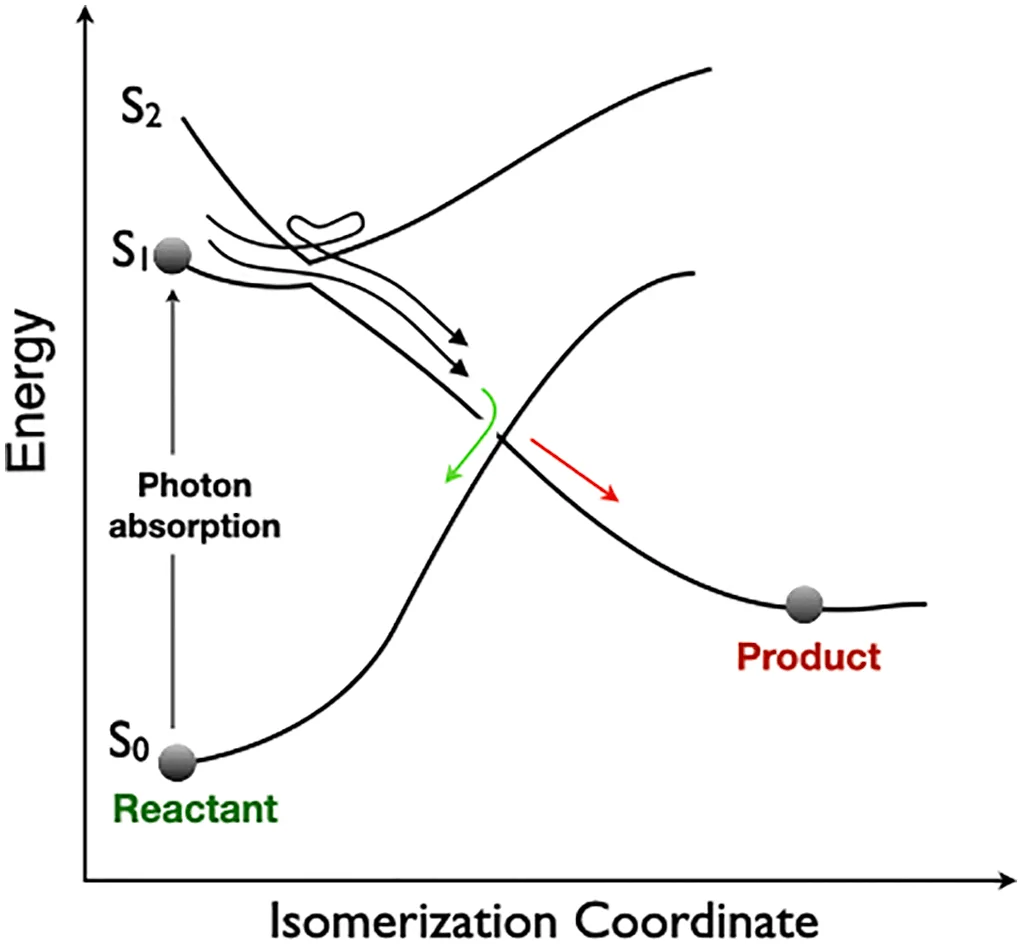

## Introduction

The protonated Schiff base of retinal (rPSB) is the chromophore of rhodopsins, a vast family of light-responsive membrane proteins found in all life domains.^1^ In spite of their large sequence diversity (*e.g.*, between microbial and animal members), rhodopsins share a common architecture characterized by a single rPSB molecule hosted in a cavity formed by seven interconnected alpha helices.^1^ Recent studies have demonstrated that artificial proteins using rPSB as their light-absorbing unit can also be synthesized in the laboratory and mimic different properties of their natural counterpart.^2–4^

Upon electronic excitation at a suitable wavelength, rPSB (see Fig. 1A, left panel) undergoes C11=C12 or C13=C14 double bond isomerization that initiates various rhodopsin functions such as vision in vertebrates and invertebrates and ion-pumping or ion-channeling in microorganisms. Remarkably, such isomerization usually occurs on a sub-picosecond time scale.^1^ However, these fast times become significantly longer when rPSB is placed in different environments. For instance, when rPSB is excited in solution (*e.g.*, in methanol), canonical spectroscopic studies show an excited state decay within 4 ps and a return to the ground state (S_0_), with photoproduct formation in 15 ps.^5,6^ Different computational studies attributed such lifetime increase to the formation of an excited state intermediate characterized by mixing between the reactive spectroscopic state dominated by a single π–π* excitation (*i.e.* S_1_) state and the dark state dominated by double (π–π*)^2^ excitation (*i.e.* S_2_).^7–10^ Such a mixing of electronic configurations would occur immediately after relaxation from the vertical excitation region (*i.e.* the Franck–Condon (FC) region). Indeed, related studies have shown that S_2_–S_1_ mixing or low-lying real crossings may be induced by different factors including the chromophore counterion, external homogeneous electrostatic fields and chemical modifications of the chromophore.^2,5,11–16^

**Fig. 1:**
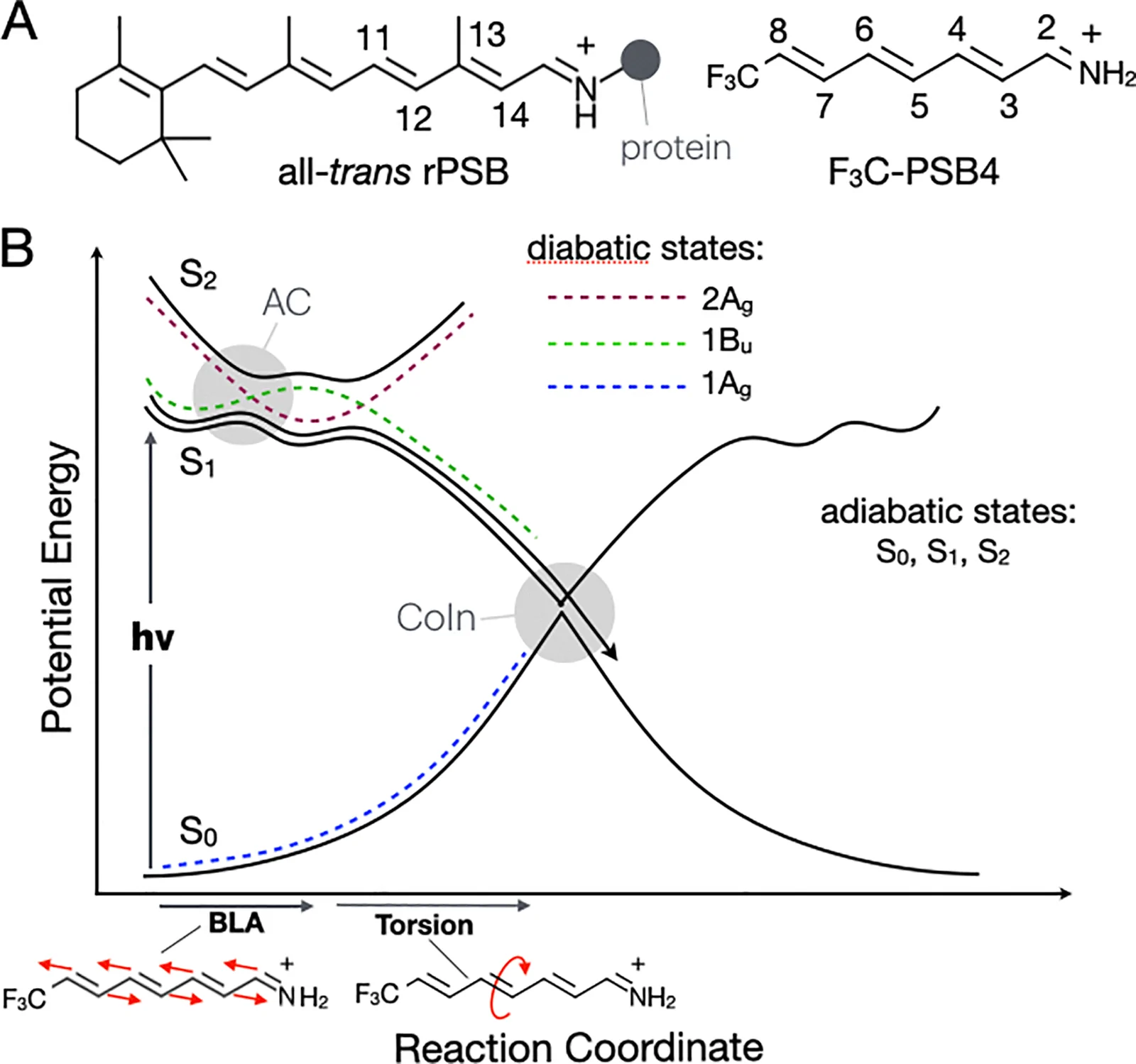
Photoisomerization mechanism for rPSB. (A) Lewis structure of the all-*trans* stereoisomer of rPSB (left panel) and of its simplified model F_3_C-PSB4 (right panel) employed here. The circle indicates the protein (opsin) covalently linked to rPSB. (B) Present isomerization mechanism. After photoexcitation, the rPSB reaches a quasi-degeneracy region between the S_2_ and S_1_ adiabatic states including one or more ACs (higher grey circle). This “motion” is followed by the isomerization motion leading to a real crossing and therefore to a CoIn (lower grey circle).

While an S_2_–S_1_ mixing was initially considered irrelevant for the rPSB photoisomerization in the rhodopsin cavity, recent computational studies on the canonical visual pigment bovine rhodopsin have shown that it affects the photoisomerization dynamics and, in principle, modulates its quantum efficiency.^17^ It has also been reported that S_2_–S_1_ mixing has been detected in microbial rhodopsins where it would explain the observation of certain excited state vibrational (*i.e.* nuclear) coherences^18^ and also provide a mechanism for increasing fluorescence emission in these typically non emissive proteins.^19,20^ These observations are important for the development of optogenetics tools where microbial rhodopsins are employed for triggering, silencing and visualizing neuronal activities.^21–23^ They also open the path to a more complete understanding of the effects of mutations on the rPSB photoisomerization efficiency and therefore on light sensitivity in rhodopsins in general, with applications in biology and medicine.

From the methodological viewpoint, the results summarized above have been obtained by combining potential energy surface (PES) mapping and quantum-classical trajectory computations (mainly FSSH trajectory propagations^24,25^ with decoherence corrections^26^). The forces driving the dynamics were computed within the Born–Oppenheimer approximation, where the electronic structure is stationary. PES mapping has shown that rPSB features a minimum energy S_2_/S_1_ conical intersection (CoIn) located close to the FC region (*i.e.* geometrically not far from the S_0_ equilibrium structure of rPSB). The region surrounding such a CoIn, or more precisely, the S_2_/S_1_ avoided crossing (AC) region, can be accessed in 10–20 fs when the initial conditions correspond, for instance, to a room temperature Boltzmann distribution (see Fig. 1B). Such initial motion is invariably described by a bond length alternation (BLA) nuclear mode (BLA is defined as the difference between the average single bond length and the average double bond length of the Lewis structure on the left panel of Fig. 1A).

The results summarized above have raised interest in what is actually happening during such a short S_1_ relaxation time not only at the nuclear, but also at the electronic level. For this reason, a recent contribution from our groups has focused on the light-induced coupled electronic and nuclear dynamics of rPSB.^27^ This required the simulation of the dynamics of the electronic wavepacket, thus allowing one to describe the electronic relaxation as a function of time. To make this possible in terms of computational costs, a simplified rPSB model (octa-3,5,7-trieniminium cation, also called Me-PSB4) was employed.

An electronic wavepacket could be created from a coherent superposition of adiabatic states. In general, this would exhibit unique properties that are different from the properties of the constituent states.^28,29^ Such a wavepacket can be constructed experimentally and studied by using laser excitation. A coherent superposition could also be created by passing through a CoIn along the direction of the derivative coupling.^30,31^ One of the authors has recently documented this effect in benzene and pyrazine.^32,33^ One can also explore the behavior of coherent superpositions using simulations that start from an “engineered” coherent superposition.^34,35^ Most relevantly for the present context, the authors have shown that by mixing S_2_ and S_1_ one can stimulate the BLA dynamics in the already mentioned Me-PSB4 model.^27^

In this work, we start by revising the results motivating the growing interest in looking at S_2_–S_1_ mixing in rPSB. We then move to revisiting the work done on Me-PSB4^27^ using electronic wavepackets and the Quantum Ehrenfest (QuEh) method.^36,37^ To effectively do so, we use a related model where the Me group is replaced by a F_3_C group (F_3_C-PSB4). Due to the presence of an electron-withdrawing group, F_3_C-PSB4 features an enhanced S_2_/S_1_ crossing probability.^16^ The QuEh method describes the nuclear motion using Ehrenfest trajectories and then couples them with electronic quantum dynamics based on Gaussian wavepackets floating on normal modes.^38,39^ We make no assumptions other than the number of normal modes that are involved in the dynamics of the S_0_, S_1_ and S_2_ electronic states. The objective is to examine whether or not the S_2_ state becomes populated in a region of sizable S_2_/S_1_ nonadiabatic coupling. As we shall see, the S_2_ state of F_3_C-PSB4 does indeed become populated on a 60 fs timescale, and, therefore, well before the system enters a second CoIn associated with the crossing between the S_2_ and the S_1_ state and ultimately leading to a decay in 100 fs.

A schematic representation of the common structure of the potential surface driving the photoisomerization of rPSB in rhodopsin and gas-phase F_3_C-PSB4 is given in Fig. 1B. Note that along the isomerization coordinate the system encounters an AC between S_2_ and S_1_ and a CoIn between S_1_ and S_0_. Also note that the reaction coordinate is multi-modal. It is dominated by a BLA mode in the part approaching the AC region. It is then dominated by double bond isomerization motion (*i.e.* a torsional mode) after such a region is abandoned to, ultimately, reach the CoIn along coordinates dominated by two coupled C11=C12 or C9=C10 double bond twisting mixing with a localized hydrogen wagging (see also below). Multimode reaction coordinates have been found in several photochemical processes driven by CoIn's.^40^

The present perspective includes two result sections. The first reviews and discusses the results achieved by constructing and studying a quantum mechanics/molecular mechanics (QM/MM) model of visual rhodopsin and its photoinduced dynamics by computing a set of a few hundred quantum-classical trajectories. The second section focuses on the previously mentioned wavepacket dynamics of F_3_C-PSB4 under isolated conditions and also discusses new results obtained using a methodology that goes beyond the Born–Oppenheimer approximation.

Before presenting the two sections mentioned above, we review the electronic character of the electronic states considered in the discussion. As shown in Fig. 1 these states are labeled using the symmetry labels for polyenes as 1A_g_, 1B_u_ and 2A_g_. At the S_0_ equilibrium geometry of rPSB, these “diabatic” states dominate the S_0_, S_1_ and S_2_ PES, respectively, with the 1B_u_ and 2A_g_ labels associated with π–π* and (π–π*)^2^ excitations, respectively. In Fig. 2 we show that the same labels are associated with Lewis's formulas displaying distinct electronic (or bonding) characters of rPSB. These correspond to a closed shell character (COV, associated with 1A_g_. This is the “covalent” character associated with the closed-shell ground state and with the homolytic breaking of the double bond *via* torsional deformation at the same time), a charge transfer character (CT, associated with 1B_u_). This can be described as the migration of one electron from the carbon end of the chromophore towards the positively charged –C=N moiety and, therefore, a simultaneous shift of the positive charge from –C=N towards the carbon end of the molecule. When a double bond is highly twisted, almost 100% of the charge resides on the moiety not containing the nitrogen and a diradical character (DIR, associated with 2A_g_. This is associated with a diradical character at the ground state equilibrium geometry and a tetraradical character in situations when one double bond is highly twisted and, therefore homolytically broken). The electronic characters mentioned above can be computationally assigned in a relatively straightforward way by calculating the positive charge distribution along the conjugated framework. Fig. 2 also demonstrates the correlation of the bonding character with the region surrounding the S_1_/S_0_ CoIn where the rPSB features a *ca.* 90° twisted double bond.

**Fig. 2:**
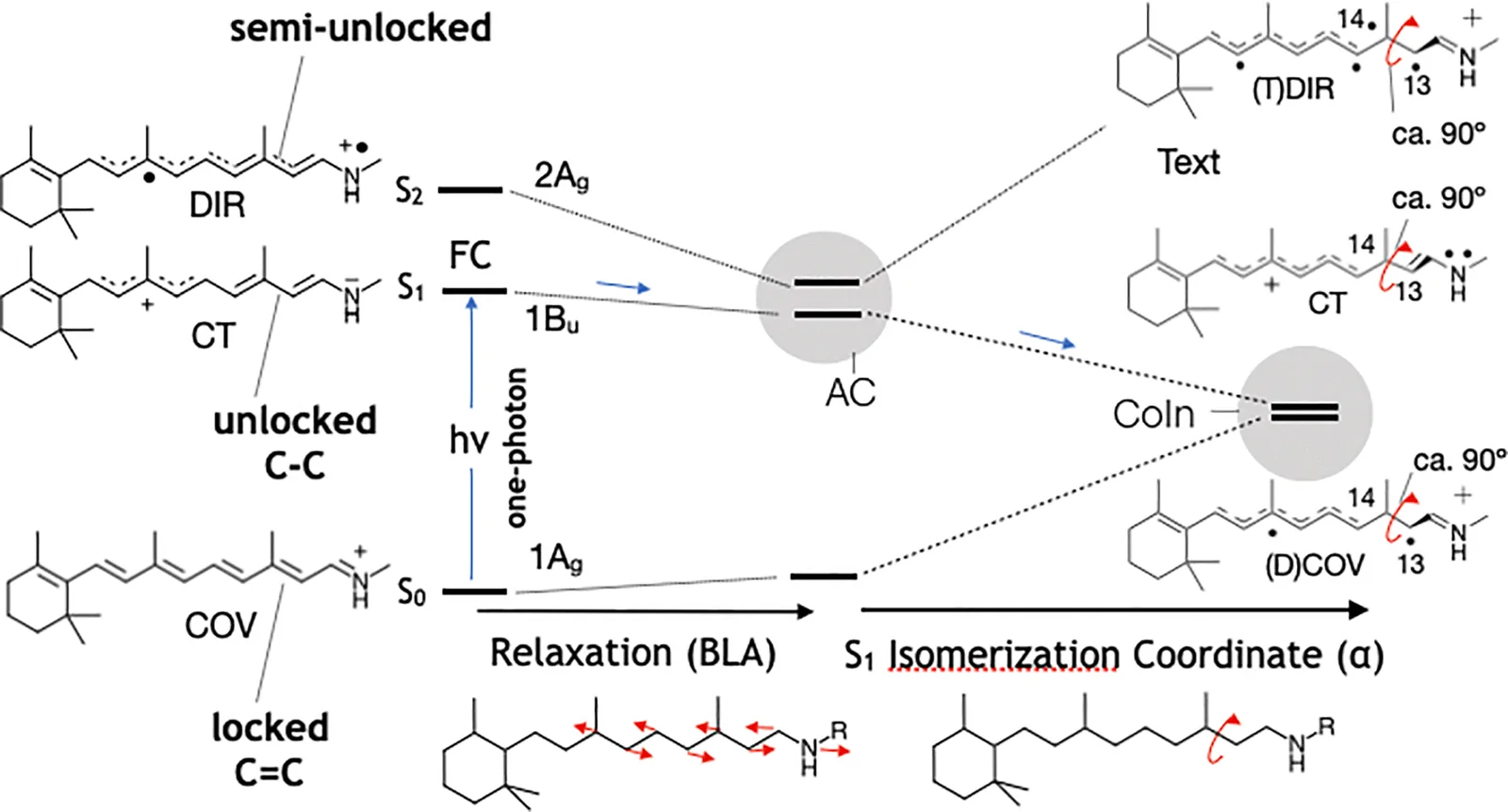
Electronic character changes along the photoisomerization coordinate of rPSB. Due to the torsional motion associated with C13=C14 twisting (*α*), the COV (1Ag or closed shell) assumes a diradical configuration. The CT state confines the positive charge on the β-ionone containing moiety upon reaching the CoIn (at *α ca.* 90°). At this same geometry, the COV character assumes a singlet diradical character ((D)COV) due to the homolytic bond-breaking. Similarly, the DIR assumes a tetraradicaloid character ((T)DIR). This figure is adapted from Fig. 1 of ref. 27. Reproduced from Fig. 1 of ref. 27 with permission from the American Chemical Society, copyright 2021.

## S_2_ and S_1_ dynamics in visual rhodopsin

The activation of rhodopsin, the light-sensitive G-protein-coupled receptor responsible for dim-light vision in vertebrates, is driven by a sub-picosecond double-bond isomerization of the 11-*cis* double bond of the rPSB chromophore, achieving a quantum efficiency of almost 70%^1^ that has been replicated in quantum-classical trajectory studies.^41^ The origin of such light sensitivity is still not completely understood. One question to be answered is whether one or more in-phase (vibrationally coherent) nuclear motions control the quantum efficiency. This question has been addressed in two relatively recent reports that we will discuss in some detail in the following subsections.

### Excited state population branching due to an S_2_/S_1_ avoided crossing region

In a recent study,^17^ we used hundreds of quantum-classical trajectories to show that, 15 fs after light absorption, a near degeneracy between S_1_ and S_2_ causes the splitting of the excited state population of bovine rhodopsin (the canonical model for vertebrate visual pigments) into subpopulations with a distinct dynamic behavior. In fact, these subpopulations propagate with different velocities and, as discussed below, lead to distinct contributions to the isomerization quantum efficiency. Such splitting is modulated by the protein electrostatics, thus linking amino acid sequence variations to quantum efficiency modulation.^17^

As documented in Fig. 3, this behavior can be explained by the presence of an S_2_/S_1_ CoIn located in the vicinity of the FC region. The geometry of a representative minimum energy structure of such a CoIn (MinCI_S_2_/S_1__) is given in Fig. 3A. As also indicated by the branching plane vectors bv_1_ and bv_2_, the MinCI_S_2_/S_1__ geometry is characterized by specific stretching distortions with respect to a typical S_0_ equilibrium structure. In fact, it features similar bond lengths in the center of the conjugated chain and a charge distribution not completely located in the reference moiety of Fig. 3B. Note that the positive charge of such a moiety informs on the electronic character of rPSB. Large values of the charge correspond to a strong CT character. Low values indicate either COV or DIR character.

**Fig. 3:**
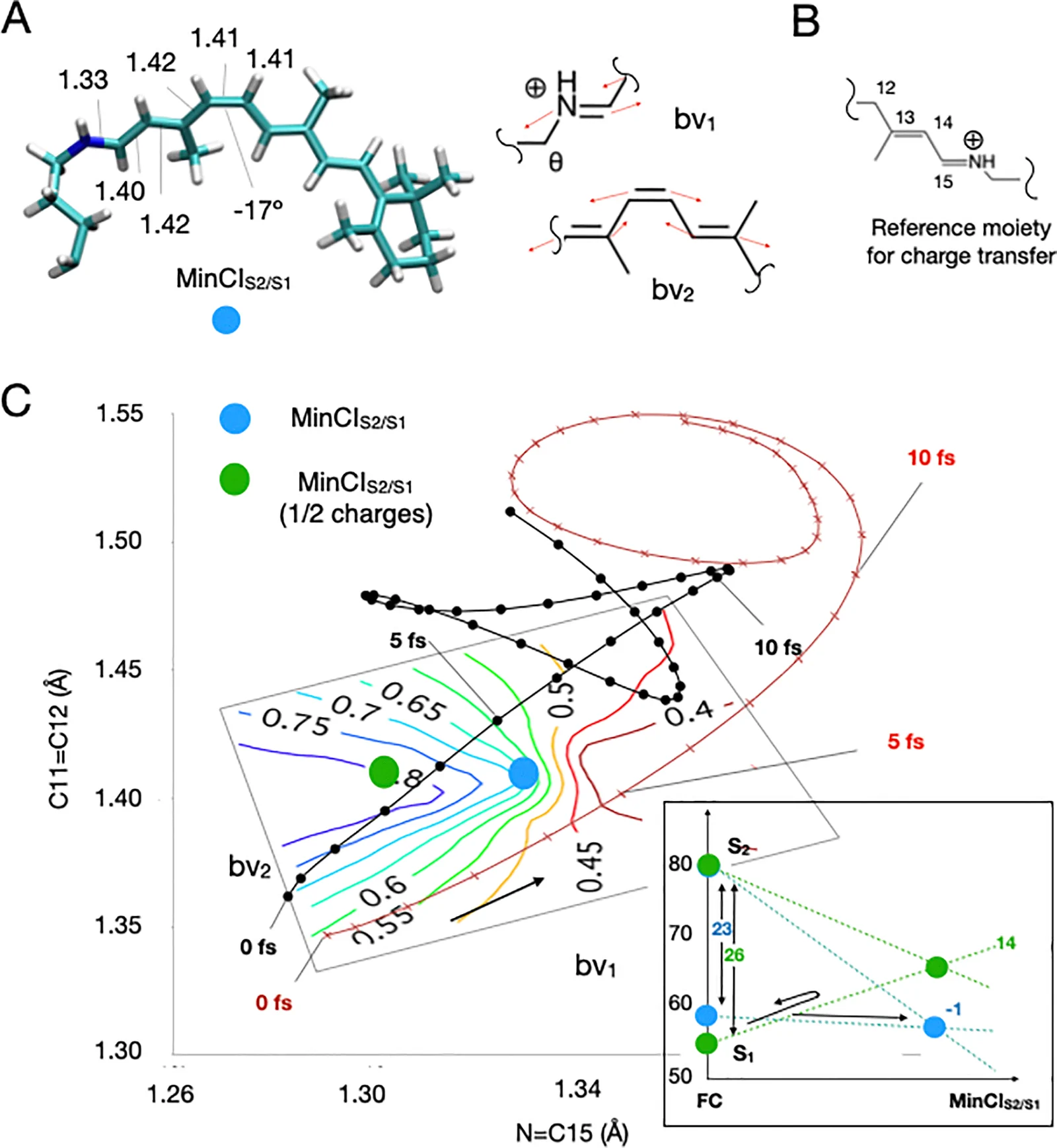
Structure of the S_1_ potential energy surface near the FC region. (A) Main geometrical parameters of the MinCI_S_2_/S_1__ of the 11-*cis* rPSB of bovine rhodopsin. The branching plane vectors are depicted on the right. (B) rPSB moiety used to determine the electronic character of different regions of the S_1_ PES. (C) Electronic character of S_1_ PES and progression along the average N=C15 and C11=C12 components of BLA. The contour diagram represents the change in the CT character plotted along the branching plane (small values represent larger CT character). The inset in the bottom right shows the effect of the environment (protein) charge reduction on the S_1_ energy differences between the MinCI_S_2_/S_1__ and the FC regions. With half charges, MinCI_S_2_/S_1__ is destabilized (from −1 to 14 kcal mol^−1^) relative to FC. Light blue circles. Adapted from Fig. 4 of ref. 17 with permission from Springer-Nature, copyright 2022.

Fig. 3C displays the geometrical changes occurring along two types of quantum-classical trajectories during the early (15 fs) motion triggered by photoexcitation. It is shown that such trajectories may traverse both a CT-like region (red trajectory) and a DIR-like region (black trajectory) roughly corresponding to the branching plane vectors. The illustration also shows that, given its semi-locked bonding character (see Fig. 2) the DIR-like region may produce trajectories that oscillate geometrically back towards the FC point and thus experience a delay. This is not the case for trajectories maintaining a CT character (trajectory in red). It is evident from the figure inspection that these types of trajectories travel on different sides of the S_2_/S_1_ CoIn. Basically, they “sense” the so-called geometric phase of the S_1_ electronic state in the CoIn vicinity. CoIn's always features a geometric phase. In the case of an S_2_/S_1_ CoIn, this manifests itself as, for instance, a change of the electronic character along a closed loop incorporating the CoIn point on the lower (S_1_) PES. When changing the geometry of the molecule along such a loop, the character of the S_1_ state may change from CT (typical of the S_1_ state) to DIR (typical of the S_2_ state) and then from DIR back to CT.

As documented in Fig. 4, it was also found that a number of the trajectories traversing the S_2_/S_1_ CoIn region undergo surface hop from S_1_ to S_2_ (*i.e.* undergo a non-adiabatic transition) and, subsequently, from S_2_ to S_1_ before entering the torsional path characterizing the isomerization coordinate and ultimately decay to S_0_*via* a highly twisted S_1_/S_0_ CoIn (see Fig. 1). Accordingly, in Fig. 4A, left panel, we display the S_1_ energy change of 200 quantum-classical trajectories representing the evolution of a “vibrational wavepacket“ produced by projecting on S_1_ a discrete representation (*i.e.* the set of geometries and velocities) of a room-temperature Boltzmann distribution. Note that the plot is limited to the first 50 fs of the wavepacket motion. Still, it is clear that *α* values close to 90 degrees and, therefore, close to an S_1_/S_0_ CoIn are reached, consistent with the reported 2D spectra and heterodyne detected transient grating methods with a higher signal-to-noise ratio that fully resolve the CoIn passage.^42,43^ A statistical representation of the S_2_/S_1_ hop points is instead given in the central panel of Fig. 4A. Finally, in the right panel, we give a schematic representation of the energy change for a trajectory that spends a certain amount of time on S_2_ before hopping back to S_1_. The hop point distribution along three different coordinates (a 3D cross section) and two different values of the torsional coordinate *α* is given in Fig. 4B. The value of *α* is proportional to the timeframes (the −10 degree) at very early times and thus before S_1_ population splitting; the larger −20 degree value is instead associated with the splitting process. It can be seen that, at early times (*α* = −10 degrees), the S_2_/S_1_ hop points of two detected fast and slow subpopulations (cross and circle symbols, respectively) display a similar distribution. At later times, during the splitting process, the slow subpopulation is located close to the regions of maximum non-adiabatic coupling (NADC).

**Fig. 4:**
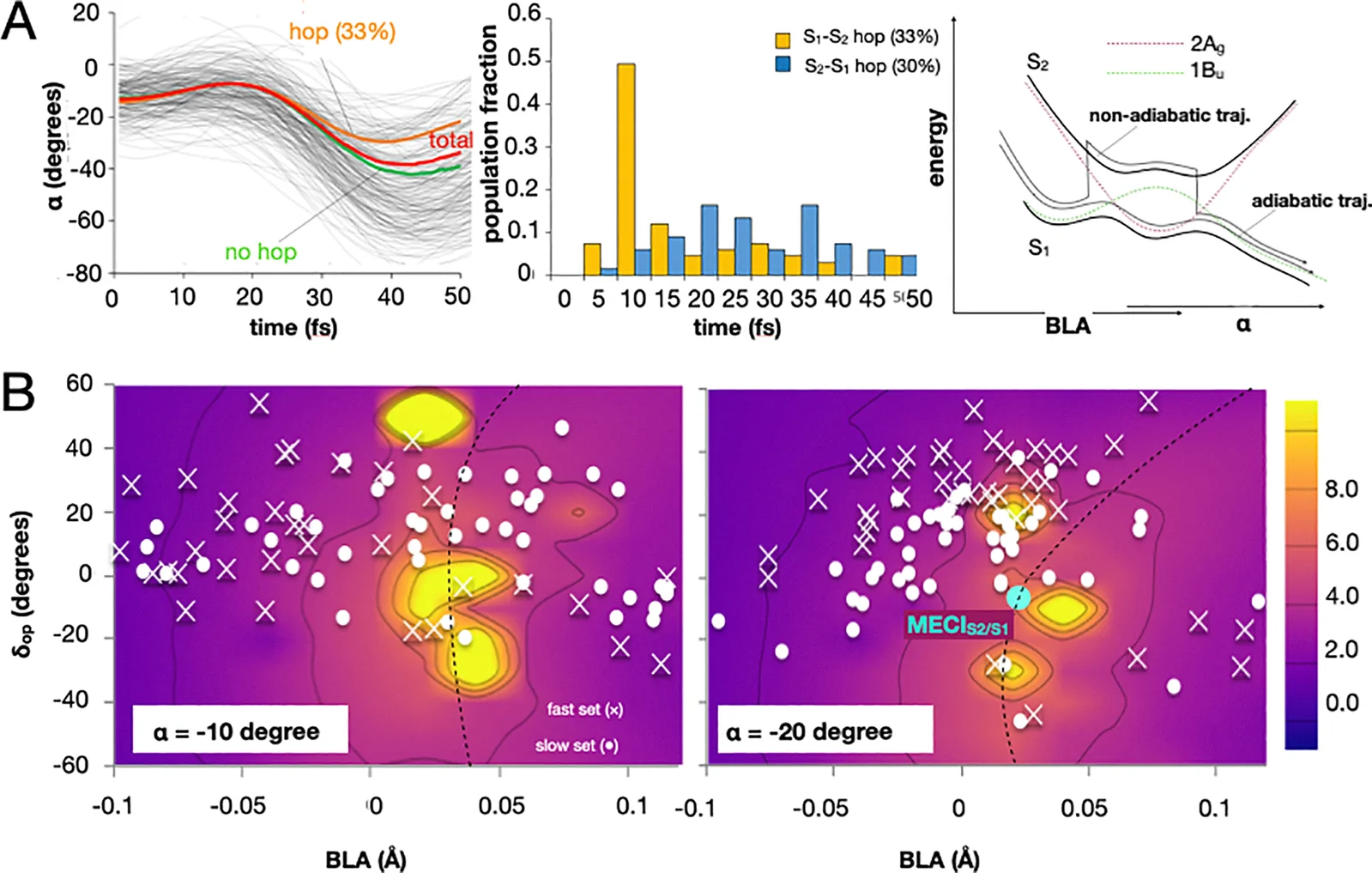
S_1_ to S_2_ and S_2_ to S_1_ hop points in the FC region of bovine rhodopsin. (A) Left panel: S_1_ energy profile along population dynamics represented by *ca.* 200 quantum-classical trajectories whose initial conditions mimic a room-temperature Boltzmann distribution. Center: Statistics of hop points. Right panel: Schematic representation of the S_2_/S_1_ non-adiabatic events (hops). (B) 3D representation of the spatial distribution of S_2_/S_1_ hops at two different values of the torsional angle alpha. The light-blue circle in the right panel corresponds to the position of the MECI_S_2_/S_1__. The *α* value corresponds to early values of the torsional coordinate. The symbol *δ*_OP_ corresponds to a vibrational mode not strictly related to the reaction coordinate and, thus, spans the intersection space. Adapted from Fig. 4 of ref. 17 with permission from Springer-Nature, copyright 2022.

In conclusion, we have located certain S_1_ trajectories that cross a region going from an initial CT reactive (in the sense of following barrierless paths leading to an S_1_/S_0_ CoIn) to a less reactive (partially locked) DIR character, resulting in slower dynamics. Another way of achieving slower dynamics is to hop from S_1_ to S_2_ and get back to S_1_ after a certain time. Such kind of trajectories have been found to emerge during the first 10–20 fs dynamics of bovine (visual) rhodopsin.

As mentioned above, the branching between slower and faster subpopulations is sensitive to the electrostatic field projected by the protein on the rPSB. Such sensitivity is evidently due to the chromophore's distinct positive charge distributions in S_2_ (with DIR character) and in S_1_ (with CT character) shown in Fig. 2. In its simplest form, such charge distribution will destabilize S_2_ and stabilize S_1_, moving the CoIn points out of the FC region.

### Impact of S_2_/S_1_ induced population splitting on the photoisomerization quantum efficiency

In the past we reported that, in an ultrafast and vibrationally coherent regime, the synchronization of specific vibrational modes is responsible for the reactivity of single trajectories in bovine rhodopsin.^41,44^ A schematic representation of a single reactive trajectory (*i.e.* one member of our quantum-classical vibrational wavepacket or population); namely a trajectory reaching the photoproduct after the S_1_ to S_0_ hop near an S_1_/S_0_ CoIn, is marked in red in the left panel of Fig. 5. More specifically, it has been found that the phase of the, so called, hydrogen-out-of-plane (HOOP) wag (*δ*_OP_), more correctly, the phase of the velocity of *δ*_OP_, must have a positive sign at the time of the S_1_ to S_0_ hop to prompt photoproduct formation. In contrast, the trajectory becomes non-reactive (*i.e.* its motion reverts back towards the reactant) when *δ*_OP_ is negative. Notice that the HOOP motion remains substantially coherent during the S_1_ propagation, with average amplitudes measured in terms of the H–C11–C12–H dihedral angle change from −35 to 28 degrees (standard deviations of 10 and 17 degrees, respectively) during the first 100 fs. Also, and more relevantly, notice that the phase of a trajectory at decay will be a function of the time it takes to reach the S_1_/S_0_ CoIn (or a point of the S_1_/S_0_ intersection space). This is clear when inspecting the schematic representation of the trajectory motion in the right panel of Fig. 5, which is based on the reaction scheme already shown in Fig. 1. Note that this behavior has been experimentally supported by observing a novel type of isotope effect.^41^

**Fig. 5:**
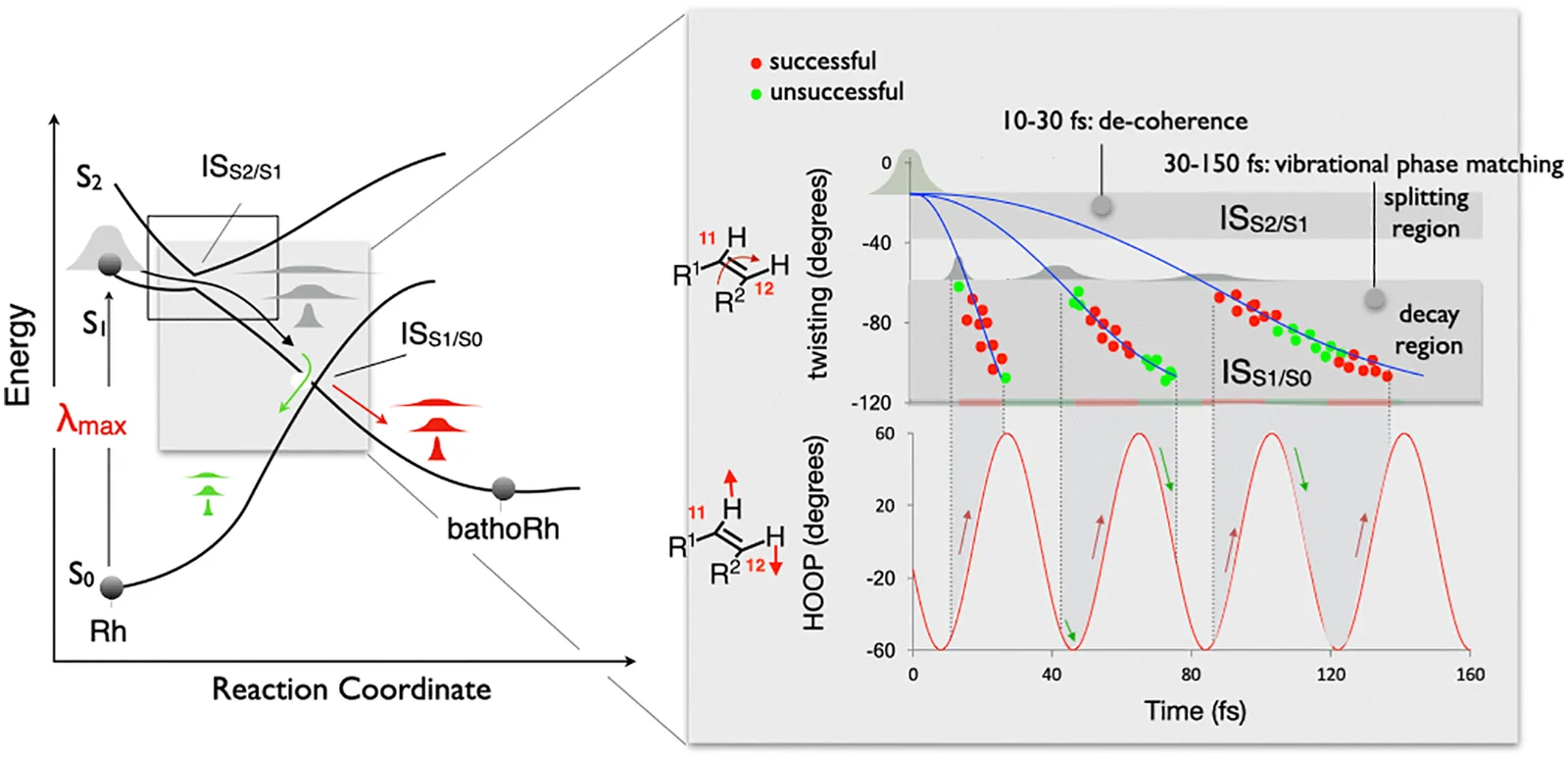
S_1_ to S_2_ and S_2_ to S_1_ hop points in the FC region of bovine rhodopsin. Left panel: S_1_ energy profile along population dynamics. Right panel: illustration of the phase relationship between isomerization motion and HOOP oscillation. Adapted from Fig. 6 of ref. 17 with permission from Springer-Nature, copyright 2022. The displayed HOOP model amplitude is close to the maximum amplitude observed in the calculation.

Since the quantum efficiency is defined by the total number of reactive trajectories, it is apparent that a fragmentation of the S_1_ population generated by light absorption, yielding subpopulations traveling with different velocities and, therefore, reaching the decay region with different average wag phases (HOOP phases), can contribute to modulating the quantum efficiency. This scenario is also illustrated in the right panel of Fig. 5. Briefly, such a phase effect refers to a change in the H–C–C–H dihedral describing the HOOP mode in the same direction as the C–C–C–C dihedral (*α*) describing the isomerization mode. The impact of the HOOP mode can be understood in terms of its effect on the overlap between the carbon p-orbitals that will eventually form the π-bond after decay.^41,44^ Anharmonic effects are assumed to be small.

Here we now recall that, due to the S_2_–S_1_ mixing effects discussed above, the initial vertically promoted and relatively compact population splits into a number of subpopulations traveling towards the S_1_/S_0_ CoIn at different velocities. This implies the total quantum yield can be computed as the sum of individual quantities, one for each subpopulation. In fact, these will reach the S_1_ to S_0_ decay point at different times and, therefore, at different values of the HOOP phase. As anticipated above, in bovine rhodopsin it has been found that an S_1_ to S_0_ decay time that coincides with a positive phase leads to an increase of quantum efficiency (see red circles). In contrast, if the time at the S_1_ to S_0_ decay has a negative sign, the subpopulation will contribute to a decrease of the quantum efficiency (see green circles). Again, note that the above computationally uncovered effect has been supported experimentally.^41^

## S_2_–S_1_ mixing and coupled electronic and nuclear wavepackets

Another and more precise way to understand the reactivity of the S_1_ state of rPSB is to consider the change in bond order generated by the CT character. As shown in Fig. 2, this features a change in the bond order which “unlocks” the double bonds, leading to a facile isomerization. The mixing with the S_2_ state of DIR character leads to a partial re-locking of the double bond, therefore, slowing down the double bond torsional deformation. Therefore, in principle, the engineering *via* shaped laser pulses (see ref. 45–48) of states with different initial S_2_/S_1_ mixing may lead to a modulation of the photoisomerization time scale and, potentially (see above), quantum efficiency. In a previous report,^27^ the authors of the present contribution have addressed exactly that problem. In fact, in that work we propagated what one could consider a “coupled electronic and nuclear wavepacket” starting with unnatural (*i.e.* engineered) electronic initial condition corresponding to a 50 : 50 superposition of the S_2_ and S_1_ states. The results demonstrated an enhancement of the double-bond single-bond (a. BLA) stretching dynamics.

In what follows, we go back to more fundamental electronic initial conditions: *i.e.* we start from a pure S_1_ state and allow the S_0_–S_1_–S_2_ mixing to evolve in a natural way, solving the time dependent Schrödinger equation (TDSE) for both electrons and nuclei (see the “Methodology” section for details). More specifically, we use the Quantum Ehrenfest (Qu-Eh) method (see below), to emulate the conditions that trigger a photochemical process. The electronic motion is described by a time dependent full wavefunction *ψ*(*r*,*t*;*R*) expressed as a linear combination of time independent electronic states with nuclear positions as parameters, and time dependent coefficients. For the nuclear motion, we select 12 normal modes from the S_0_ PES as a basis, which were chosen to span stretches, hydrogen-out-of-plane modes, bends, and torsions (again, see the “Methodology” section). A Gaussian wavepacket (GWP) *g*_*i*_(*R*,*t*) with *i* = 1, 12 is then associated with each selected normal mode.

At time zero, a chosen *g*_*i*_(*R*,*t*), representing the initial molecular population, is promoted to S_1_ vertically (GWP1). From there, this population would relax and mix with the 12 normal modes (with initial momentum) GWPs (GWPn). Such a relaxation is driven by the gradient determined by the S_0_, S_1_ and S_2_ non-adiabatic mixing. Therefore, each GWP moves on its own time-dependent PES with a specific mixed electronic character. These normal mode associated GWPs, with their mixed electronic characters, will then mix during the propagation.

The main results are contained in the population “snapshots” of Fig. 6–9 (in the legends, CI means Configuration Interaction and reflects the weights of the three electronic states in the *ψ*(*r*,*t*;*R*)) taken during the time frame where the states are strongly mixed. The representative geometries have been derived by taking a representative approximate midpoint in the region of strong mixing. While these results cannot offer a complete description of the F_3_C-PSB4 isomerization dynamics, they can be used to prove that the three electronic states mix. More specifically, the plotted data demonstrate that S_2_ becomes populated in the region of S_2_/S_1_ AC. Together with the result reported in ref. 27, our results predict that state mixing arises even in a normal photochemical experiment as well as an “engineered” superposition. In the following, we focus on the detailed analysis of the electronic state evolution initiated along a few selected normal modes and GWPs. In fact, a variety of different electronic mixing situations are shown to be possible.

**Fig. 6:**
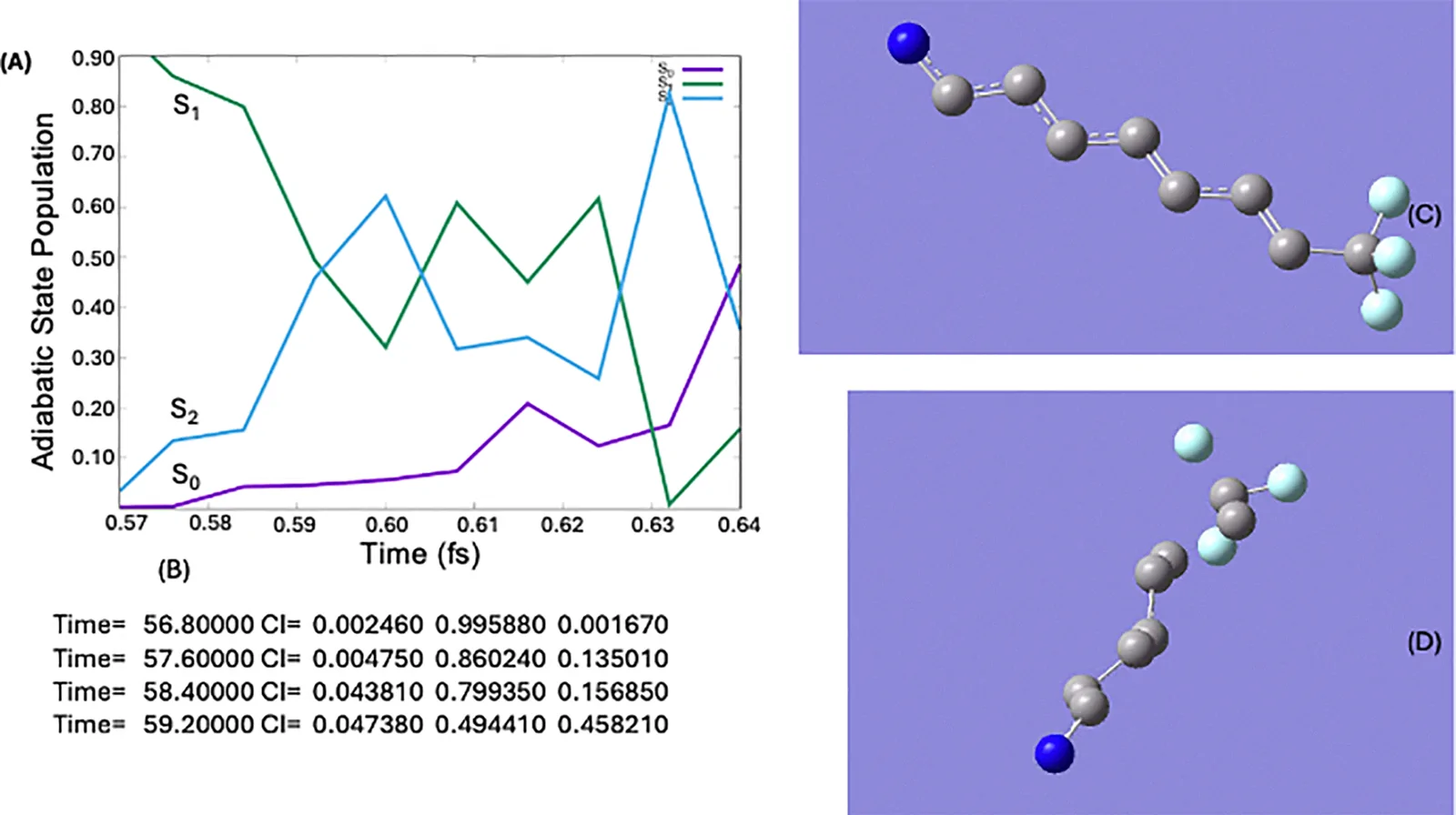
S_0_–S_1_–S_2_ state propagation initiated on normal mode 6 (GWP 2, delocalized bond twisting) showing the populations of the adiabatic states in the region of S_2_–S_1_ mixing. (A) Populations of S_0_, S_1_ and S_2_. Note the emergence of the mixed state between 57 and 63 fs before decay to S_0_. (B) Numerical populations in the 56–58 fs range. (C) and (D) Snapshots of geometries (these are extracted from the approximate middle strongly mixed region shown in panel B) of the figure in region of the S_2_–S_1_ mixing showing stretching (C) and torsion (D).

**Fig. 7:**
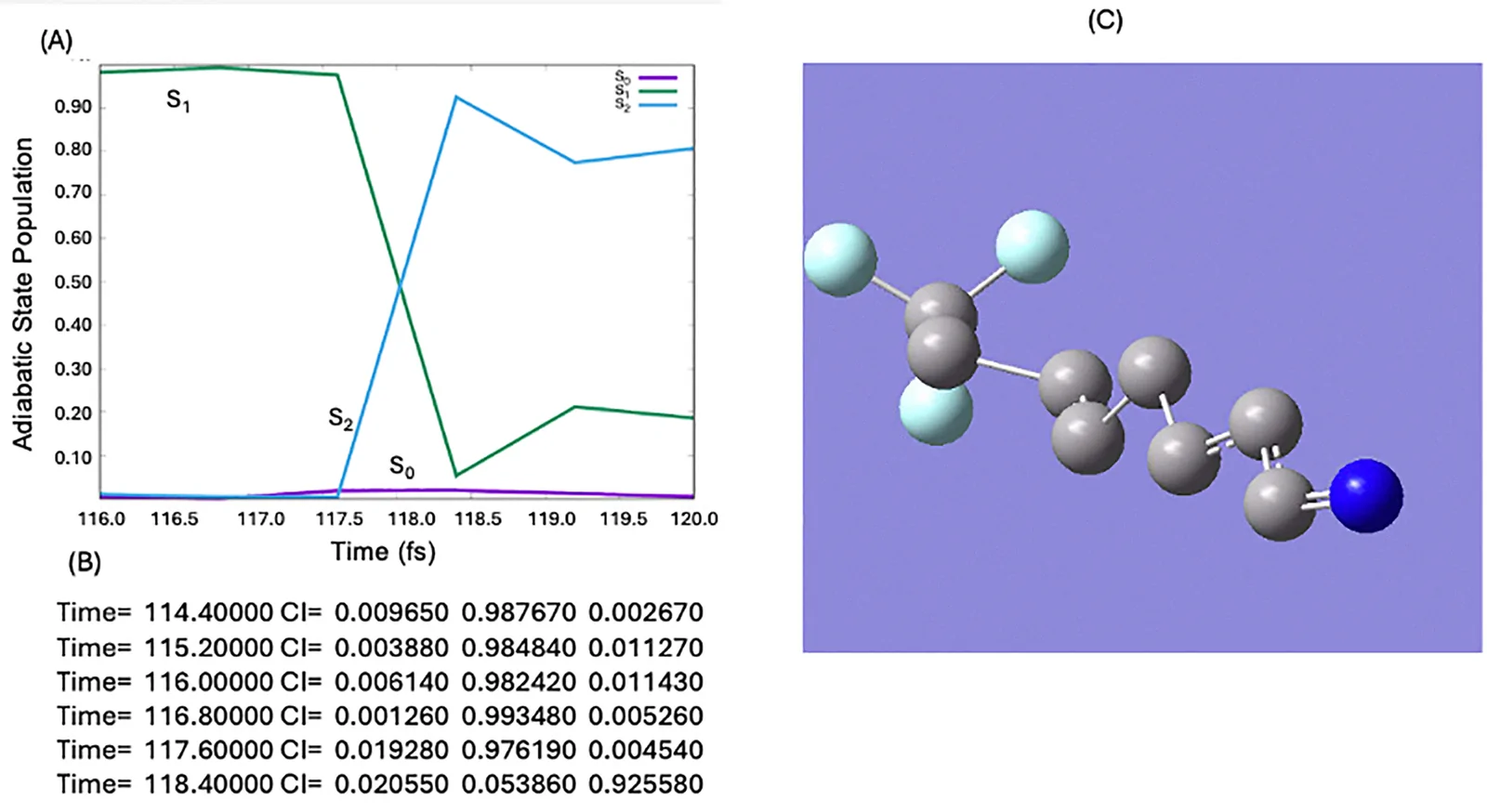
S_0_–S_1_–S_2_ state propagation initiated on normal mode 10 (GWP 4, delocalized bond twisting) in the region of S_2_–S_1_ mixing. Note that S_2_ becomes populated. (A) Populations of S_0_, S_1_ and S_2_. (B) Populations. (C) Geometry (this is extracted from the approximate middle strongly mixed region shown in panel B of the figure) in the region of S_2_/S_1_ crossing showing stretching and torsions.

**Fig. 8:**
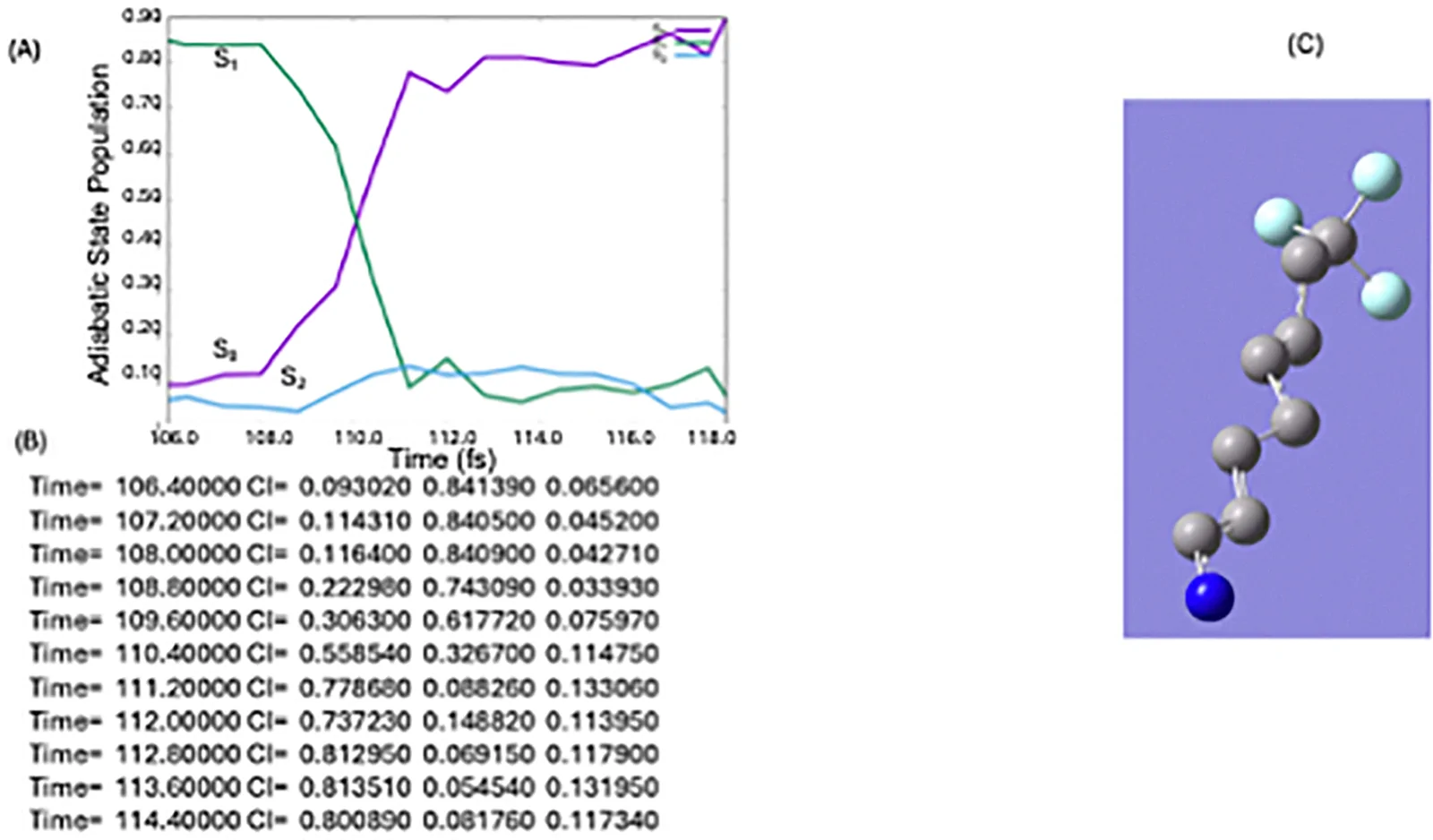
S_0_–S_1_–S_2_ state propagation initiated on normal mode 22 (GWP 9, delocalized H out-of-plane). Populations S_0_–S_1_–S_2_ in the region of S_1_–S_2_ mixing. (A) Populations S_0_ S_1_ S_2_. (B) Numerical population and (C) geometry (this is extracted from the approximate middle of the nuclear density plot) in the region of S_1_–S_2_ mixing: stretching and torsion.

**Fig. 9:**
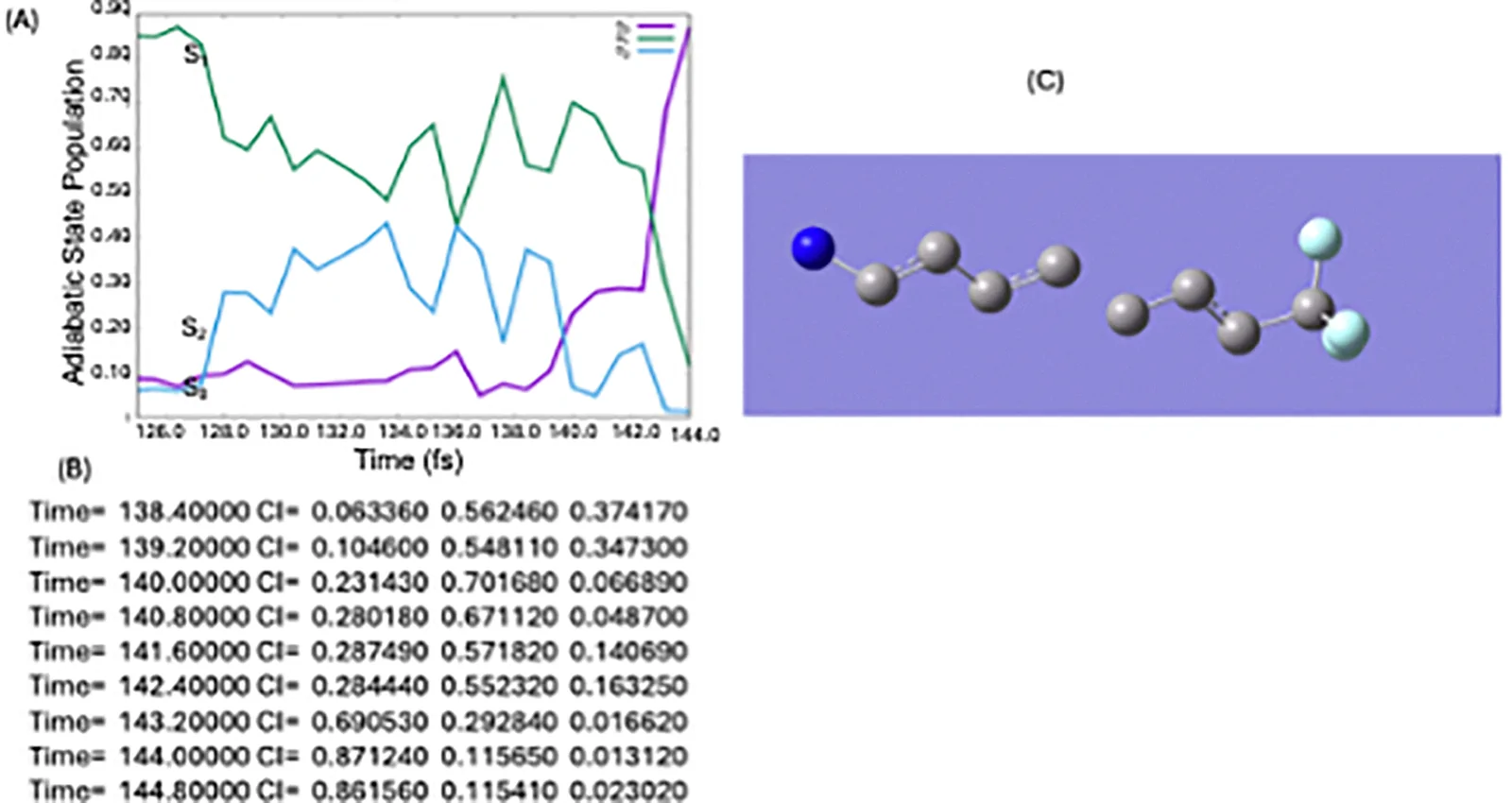
Normal mode 24 (GWP 1, delocalized H out-of-plane bending) populations S_0_, S_1_, and S_2_ in the region of S_0_–S_1_–S_2_ mixing. (A) Populations S_0_, S_1_ and S_2_. (B) Numerical population. (C) Geometry (this is extracted from the approximate middle strongly mixed region shown in panel B of the figure) in the region of S_0_–S_1_–S_2_ mixing: stretching and torsion.

In Fig. 6 and 7 we illustrate population dynamics patterns for S_2_–S_1_ mixing starting from the pure S_1_ state and after initiating the nuclear motion on mode 6 and mode 10 of S_0_ (see above), respectively. Fig. 6 shows continuous mixing during the 50–60 fs time segment. In contrast, in Fig. 7 we show that the production of a longer lived S_2_ state is also possible. In Fig. 8 we show instead a case where there is a single S_1_/S_0_ decay without generation of an S_2_/S_1_ mixed state. Taken together, Fig. 6–8 show three qualitatively different mechanistic possibilities.

In Fig. 9 and 10 we report the same data starting from other normal modes. In Fig. 9 we first observe a slower S_2_–S_1_ mixing in the 130–140 fs time segment followed by S_1_ to S_0_ decay. Finally, in Fig. 10 we observe a case where the S_1_ and S_0_ states remain mixed for a long time before decay to S_0_.

**Fig. 10:**
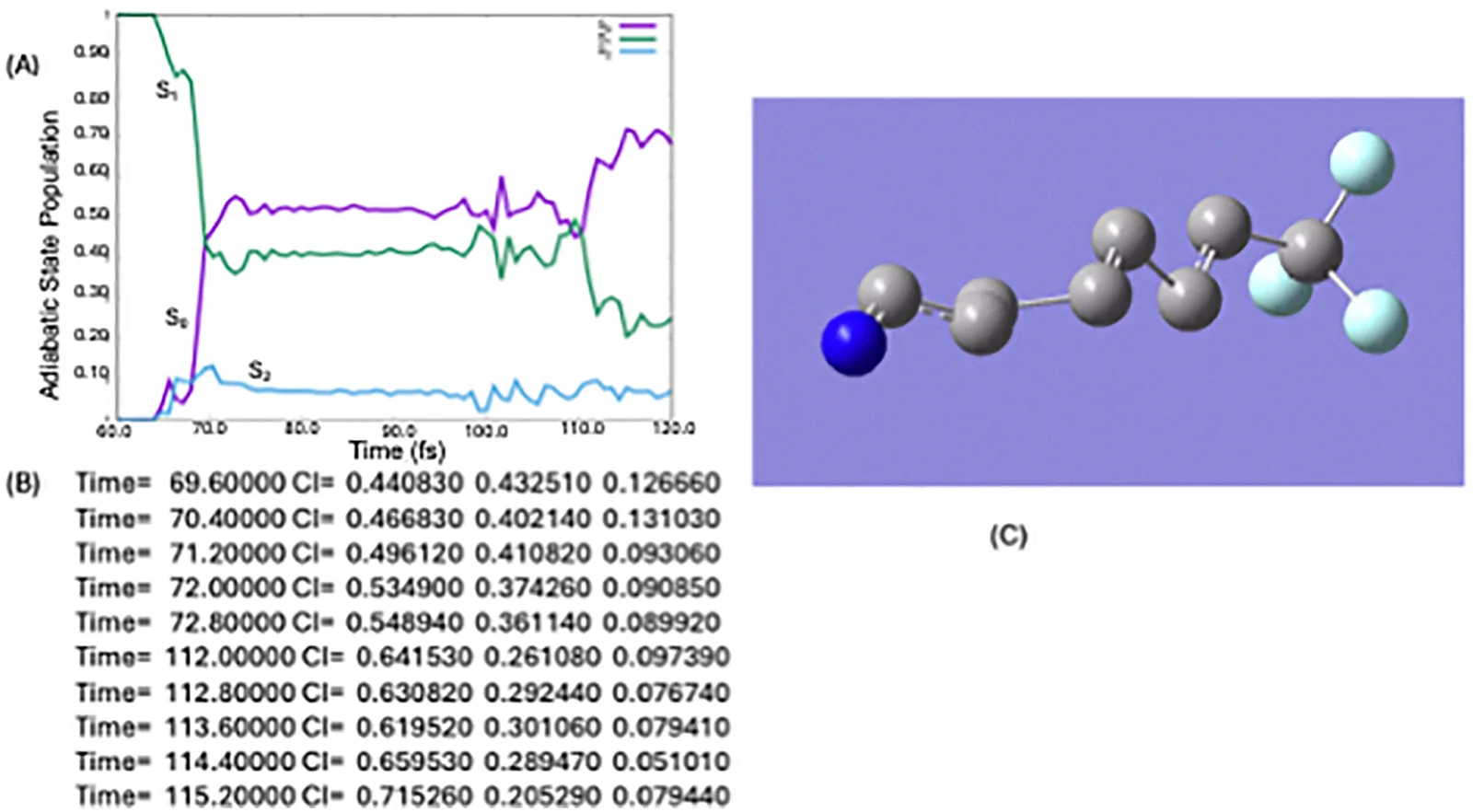
S_0_–S_2_ state propagation initiated on normal mode 16 (GWP 7, delocalized C–C–C). Populations S_0_–S_1_–S_2_ in the region of S_0_–S_2_ mixing. (A) Populations S_0_, S_1_, S_2_. (B) Numerical population. (C) Geometry (this is extracted from the approximate middle strongly mixed region shown in panel B) of the figure in the region of S_1_–S_2_ mixing (70 fs stretching and torsion).

We should emphasize that in Fig. 7–10 we show snapshots of the state populations only where strong mixing occurs after the computations were initiated on a pure S_1_ state. One can see that the state transition does not occur “instantly” (as assumed to occur in the surface hop method), but rather, one sees propagation on an intermediate mixed state. In the latter, the mixed state gradient is that of the superposition as a whole and is not simply related to the gradient of either state on its own.

## Concluding remarks

We have demonstrated that three electronic state molecular dynamics started at the FC point on S_1_ for a PSB model populates S_2_ at intermediate times before ultimate decay to S_1_ at longer times. Furthermore, there is evidence of propagation on a coherent superposition of S_1_ and S_2_ at various times. Inspection of molecular geometries at intermediate times shows strong mixing of torsion, skeletal deformation and out-of-plane modes. This suggests that in the intermediate AC region, propagation on mixed S_2_/S_1_ states may be common. Of course, such dynamics cannot be treated with a single quantum-classical trajectory method since the crossing is not localized.

To confirm the existence of S_2_–S_1_ mixed states, we need to perform quantum dynamics. More specifically, we require dynamics where electronic and nuclear motions are coupled. Furthermore, as we have mentioned above, the normal modes/trajectories are coupled in QuEh. This has the advantage that one may get useful chemical information with a smaller number of trajectories. However, the details of a trajectory initiated on a particular normal mode will depend on the number of normal modes chosen at the outset. Thus, for example, in Fig. 6, we see a decay pattern with a GWP initiated on mode 6. However, at 58 fs, all modes are strongly mixed. Furthermore, with a different set of normal modes, this decay pattern might be seen in a different GWP. So, the obtained electronic structure evolution for F_3_C-PSB4 is correct under the assumption that the set of normal modes chosen spans the space of the S_1_–S_2_–S_3_ coupling adequately.

## Methodology

The methodology related to the first result (*i.e.* the quantum-classical dynamics of bovine rhodopsins) is fully described in ref. 26. Briefly, the QM/MM model of the visual rhodopsin was constructed from the 2.2 Å resolution crystallographic structure (Protein Data Bank ID: 1U19) of bovine rhodopsin. The retinylidene chromophore, NH group and C_ε_H_2_ atoms linked to the C_δ_ atom of the Lys296 side chain form the QM layer. The rest of the protein forms the MM layer, described by a modified AMBER94. All side chains and water molecules with at least one atom within 4 Å of any atom of the retinal chromophore were kept flexible, while all remaining atoms were kept frozen. We propagated 200 quantum–classical trajectories starting from the 200 initial conditions (consistent with a room temperature Boltzmann distribution) for 200 fs each at the two-root-state-average CASSCF/6-31G*/AMBER level using the Tully surface-hop method and equal S_0_ and S_1_ weights. The trajectories yielded a simulation of the dynamics of the entire population. To study the impact of surface hopping on the initial 50 fs dynamics describing population splitting, the full trajectory set was recomputed at both the three-root-state-average CASSCF/6-31G*/AMBER level with equal S_0_, S_1_ and S_2_ weights as well as with 0.0, 0.5 and 0.5 weights (equivalent to a two-root-state-average CASSCF/6-31G*/AMBER level with equal S_1_ and S_2_ weights). All calculations were performed with the Molcas-Tinker Interface, also available in the OpenMolcas package.^49^

The Qu-Eh method treats both the electrons and nuclei quantum mechanically *via* the TDSE. The nuclear-electronic wavefunction has the form:^36^1
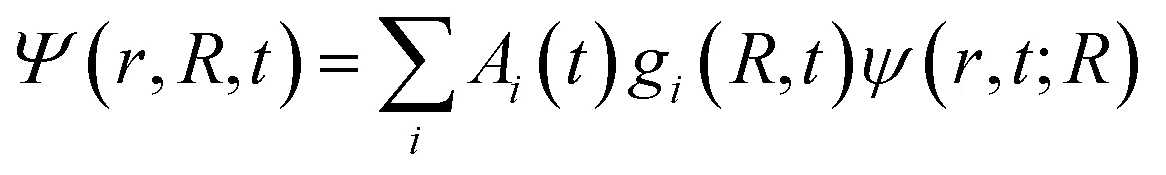
where *A*_*i*_(*t*) are the expansion coefficients for the GWPs *g*_*i*_(*R*,*t*) and are Gaussian wavepackets initiated on selected normal modes. From a conceptual (*i.e.* practical/computational) point of view, the centroid of each GWP, *g*_*i*_(*R*,*t*), moves along a “slice”, *i* (*i* labels a normal mode), of the Ehrenfest potential. The time dependent electronic wavefunction projected onto this slice, *ψ*^*i*^(*r*,*t*;*R*), is expanded as a linear combination of many-electron time-independent basis states, *ϕ*^*i*^_*j*_(*r*;*R*), with coefficient *c*^*i*^_*j*_(*t*), varying in time:2
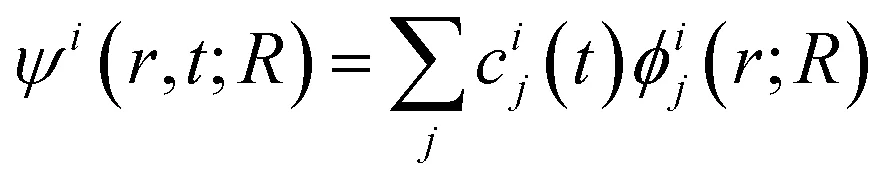
Their respective expansion coefficients, *c*^*i*^_*j*_(*t*), represent the electronic wavefunction CI coefficients, and when practically propagating the electronic wavefunction in discretized time in terms of one obtains:

3 *C*^*i*^(*t*_*k*_) = exp(−*iĤ*^*i*^(*t*_*k*_)*t*)*C*^*i*^(*t*_*k*−1_)

Note that, in practice, the basis states, *ϕ*^*i*^_*j*_(*r*;*R*), are not strictly time independent and the orbitals advance by a quadratic step for each clock tick.

The potential and wavefunction along this Ehrenfest “slice” are very similar to the equations used in the surface hopping method. We have discussed this relationship in a pedagogical form in a book chapter.^50^ In surface hopping eqn (3) is still used, but the coefficients contained within are used to determine the hop probability and the computation proceeds on an adiabatic state. In QuEh we compute the gradients and second derivatives,^37^ and *C*^*i*^(*t*_*k*_) defines the Ehrenfest potential along the slice *i*. Once propagated, CAS-CI is used to evaluate the energy, gradient, and Hessian of the Ehrenfest wavefunction (the gradient and Hessian are computed using the methods of Almlöf and Taylor^51^). An approximate local potential surface can then be constructed *via* the local harmonic approximation (LHA), which is then used to construct the nuclear Hamiltonian, which propagates the equations of motion for the nuclei.

In practice, one must select a subset of normal modes in which to carry out the computations. In our F_3_C-PSB4 model, there are some 54 normal modes. In this work, we are interested in the torsions, skeletal vibrations and out-of-plane motions that have been found to couple the electronic states in other work. We carried out preliminary computations in the 23 normal mode set. We then chose 12 normal modes from that set where we observed an S_1_ → S_2_ population change. Thus, we have chosen 6 mainly torsional modes (nm 6, 8, 10, 11, 14, 17), obtained from a b3lyp/6-31g* ground state frequency computation, and 6-combination skeletal out-of-plane modes (20, 22, 25, 25, 26, 28). However, such a selection is not too critical. In QuEh, all the modes are coupled, and the momentum flows from one mode to another, and the modes mix within a GWP; our objective was simply to look for the involvement of S_1_–S_2_ mixing before ultimate decay to S_0_ at longer times. In the end, we can demonstrate/illustrate the role of S_1_/S_2_ coupling with 5 normal modes that we have used in Fig. 7–10.

All computations were carried out with a development version of Gaussian^52^ and Quantics.^53^

## Author contributions

Both MO and MAR designed and wrote the manuscript. MO reviewed the bovine rhodopsin in the first results section (S_2_ and S_1_ dynamics in a visual rhodopsin). MAR designed, performed the calculations and wrote the F_3_C-PSB4 section (S_2_–S_1_ mixing and electronic wavepackets). Both authors wrote the Methodology and the Concluding remarks sections.

## Conflicts of interest

There are no conflicts to declare.

## Data Availability

Data from previous work can be found in the supplementary information of ref. 27. Data for Fig. 6–10 can be obtained from the authors.
